# Novel frameshift mutation in *LIS1* gene is a probable cause of lissencephaly: a case report

**DOI:** 10.1186/s12887-022-03595-6

**Published:** 2022-09-14

**Authors:** Camila Simoes, Martín Graña, Soledad Rodriguez, Federico Baltar Yanes, Alejandra Tapié, Nicolás Dell’Oca, Hugo Naya, Víctor Raggio, Lucía Spangenberg

**Affiliations:** 1 Bioinformatics Unit, Institut Pasteur de Montevideo, Mataojo 2020, 11400 Montevideo, Uruguay; 2 Biological Engineering Group, CENUR Litoral Norte, Universidad de La República, Paysandú, Uruguay; 3 Departamento de Genética, Facultad de Medicina, Universidad de La República, Montevideo, Uruguay; 4 Cátedra de Neuropediatría, Facultad de Medicina, Centro Hospitalario Pereira Rossell, Universidad de La República, Montevideo, Uruguay; 5 Departamento de Producción Animal y Pasturas, Facultad de Agronomía, Universidad de La República, Montevideo, Uruguay; 6 Departamento Básico de Medicina, Hospital de Clínicas, Universidad de la República, Montevideo, Uruguay

**Keywords:** Lissencephaly, *PAFAH1B1*, Whole-genome sequencing, Rare disease, Novel mutation, Case report

## Abstract

**Background:**

Lissencephaly (LIS) is a cortical malformation, characterized by smooth or nearly smooth cerebral surface and a shortage of gyral and sulcal development, which is caused by deficient neuronal migration during embryogenesis. Neuronal migration involves many gene products, among which is the product of the *PAFAH1B1* gene, associated with this disease. LIS is a rare disease, characterized by low population frequency, and with non-specific clinical symptoms such as early epilepsy, developmental delay or cerebral palsy-like motor problems. Given that high-throughput sequencing techniques have been improving diagnosis, we have chosen this technique for addressing this patient.

**Case presentation:**

We present the case of a seven years old male patient with an undiagnosed rare disease, with non-specific clinical symptoms possibly compatible with lissencephaly.

The patient was enrolled in a study that included the sequencing of his whole genome. Sequence data was analyzed following a bioinformatic pipeline. The variants obtained were annotated and then subjected to different filters for prioritization. Also mitochondrial genome was analyzed. A novel candidate frameshift insertion in known *PAFAH1B1* gene was found, explaining the index case phenotype. The assessment through in silico tools reported that it causes nonsense mediated mechanisms and that it is damaging with high confidence scores. The insertion causes a change in the reading frame, and produces a premature stop codon, severely affecting the protein function and probably the silencing of one allele. The healthy mother did not carry the mutation, and the unaffected father was not available for analysis.

**Conclusions:**

Through this work we found a novel de novo mutation in *LIS1/PAFAH1B1* gene, as a likely cause of a rare disease in a young boy with non-specific clinical symptoms. The mutation found correlates with the phenotype studied since the loss of function in the gene product has already been described in this condition. Since there are no other variants in the *PAFAH1B1* gene with low population frequency and due to family history, a de novo disease mechanism is proposed.

**Supplementary Information:**

The online version contains supplementary material available at 10.1186/s12887-022-03595-6.

## Background

Lissencephaly (LIS) is a subtype of malformations of cortical development (MCD), which are a heterogenous group of disorders with diverse phenotypic and genotypic presentations. Patients with LIS may present different degrees of developmental delays, seizures, severe psychomotor impairment, muscle spasticity or hypotonia [1].

Lissencephaly is a disorder caused by a defect in neuronal migration, which occurs between 12 and 24 weeks of gestation and results in a lack of development of brain folds (gyri) and grooves (sulci) [2]. Neuronal migration is a complex process, which requires the coordination of many gene products.

*LIS1* and *DCX* were the first genes that were associated with LIS, discovered in 1993 and 1998, respectively [3, 4]. In the past years with the advent of new molecular genomics technologies, many additional genes were found. These LIS-related genes include *ACTB, ACTG1, ARX, CDK5, CRADD, DYNC1H1, KIF2A, KIF5C, NDE1/NDEL1, TUBA1A, TUBA8, TUBB, TUBB2B, TUBB3, TUBG1, RELN and VLDLR*. Many of these 19 LIS-associated genes are related to microtubule structural proteins (tubulin) or microtubule-associated proteins [5].

The *PAFAH1B1* gene (Genbank accession number: NM_000430), located at chromosome 17p13.3, encodes the alpha subunit of the 1B isoform of the platelet-activation factor acetylhydrolase regulatory, a highly conserved protein of 410 amino acids, known as LIS1 or PAFAH1B1 [6]. It has two protein coding transcripts and several non-coding ones. LIS1/PAFAH1B1 forms the non-catalytic subunit of the G protein-like heterotrimeric cytosolic platelet-activating factor acetylhydrolase (PAF-AH) brain isoform Ib (PAFAH1B1) [7]. Along with two other subunits, PAFAH1B2 and PAFAH1B3, LIS1/PAFAH1B1 forms a trimeric complex which regulates the level of platelet activating factor (PAF) in the brain, by catalyzing the removal of the acetyl group at the SN-2 position of platelet-activating factor [8, 9]. The regulation of optimal concentrations of PAF in the brain may be critical for correct neuronal migration, essential for normal brain development and function. LIS1/PAFAH1B1 has also been shown to play a central role in the organization of the cytoskeleton, which in turn affects neuronal proliferation and migration [6]. Mutations in this gene have previously been associated with cortical brain malformation in children (Table 1).

**Table 1:** Mutations in *LIS1/PAFAH1B1* gene associated with cortical brain malformation available in ClinVar [10]. All mutations are associated with lissencephaly phenotype but two that are marked with *(associated with Subcortical band heterotopia) and ** (associated with abnormal cortical gyration)

Name	Protein change	Mutation type	Accession	GRCh37Location	dbSNP ID
**Likely Pathogenic**					
c.1142A > G (p.His381Arg)	H381R	missense	VCV000931583	2,583,597	rs2069361452
c.967 T > A (p.Trp323Arg)	W323R	missense	VCV000812182	2,579,865	rs2069271269
c.900G > C (p.Glu300Asp)	E300D	missense	VCV000436141	2,577,582	rs587784291
c.121G > A (p.Glu41Lys)	E41K	missense	VCV000159503	2,569,313	rs587784250
c.503G > A (p.Cys168Tyr)	C168Y	missense	VCV000159525	2,573,560	rs200390886
c.569-3del		non coding	VCV000211827	2,575,943	rs797045863
c.671 + 4A > G		non coding	VCV000159536	2,576,055	rs587784279
c.671 + 5G > A		non coding	VCV000159537	2,576,056	rs587784280
c.751A > C (p.Ser251Arg)	S251R	missense	VCV000159545	2,577,433	rs587784287
c.900G > A (p.Glu300 =)		missense	VCV000159550	2,577,582	rs587784291
c.938C > T (p.Ser313Phe)	S313F	missense	VCV000159552	2,579,836	rs587784293
c.965 T > G (p.Met322Arg)	M322R	missense	VCV000159553	2,579,863	rs587784294
c.1193G > A (p.Gly398Asp)	G398D	missense	VCV000159500	2,585,056	rs587784247
NC_000017.11:g.(?_2638238)_(2638345_?)del		large deletion	VCV000495279	2,541,532—2,541,639	
NC_000017.11:g.(?_2680139)_(2681852_?)del		large deletion	VCV000495278	2,583,433—2,585,146	
c.899A > G (p.Glu300Gly)	E300G	missense	VCV000436140	2,577,581	rs1555527149
c.400-1G > A		splicing acceptor	VCV001526061	2,573,456	
c.661G > A (p.Val221Met)	V221M	missense	VCV000931348	2,576,041	rs1262666760
c.1009C > G (p.His337Asp)	H337D	missense	VCV000159488	2,583,464	rs587784236
c.1190C > T (p.Thr397Ile)	T397I	missense	VCV000429277	2,585,053	rs1131691295
c.722G > C (p.Arg241Pro)*	R241P	missense	VCV000008080	2,577,404	rs121434488
**Pathogenic**					
c.441dup (p.Gly148fs)	G148fs	frame shift	VCV000211825	2,573,495—2,573,496	rs797045861
c.162dup (p.Trp55fs)	W55fs	frame shift	VCV000021181	2,569,346—2,569,347	rs113994198
c.770_772delinsTGACCCA (p.Thr257fs)	T257fs	frame shift	VCV000211832	2,577,452—2,577,454	rs797045868
c.716dup (p.Met239fs)	M239fs	frame shift	VCV000159542	2,577,397—2,577,398	rs587784284
c.1050del (p.Lys351fs)	K351fs	frame shift	VCV000021176	2,583,500	rs113994200
c.703_704del (p.Glu235fs)	E235fs	frame shift	VCV000211829	2,577,382—2,577,383	rs797045865
c.3G > A (p.Met1Ile)	M1I	missense	VCV000159520	2,541,585	rs587784265
c.33-3C > T		non coding	VCV000159514	2,568,663	rs587784260
c.37C > T (p.Arg13Ter)	R13*	stop gain	VCV000159516	2,568,670	rs587784262
c.56 T > G (p.Leu19Arg)	L19R	missense	VCV000159529	2,568,689	rs587784272
c.71_72dup (p.Glu25fs)	E25fs	frame shift	VCV000211830	2,568,702—2,568,703	rs797045866
c.72 T > G (p.Tyr24Ter)	Y24*	stop gain	VCV000159543	2,568,705	rs587784285
c.84 T > G (p.Tyr28Ter)	Y28*	stop gain	VCV000159547	2,568,717	rs369259961
c.136_137del (p.Lys46fs)	K46fs	frame shift	VCV000159505	2,569,325—2,569,326	rs587784252
c.152del (p.Leu51fs)	L51fs	frame shift	VCV000159506	2,569,341	rs587784253
c.190_192 + 5dup		splice donor	VCV000211820	2,569,381—2,569,382	rs797045857
c.192G > C (p.Lys64Asn)	K64N	missense	VCV000159511	2,569,384	rs587784257
c.192 + 1G > T		splice donor	VCV000159510	2,569,385	rs587784256
c.192 + 1G > A		splice donor	VCV000159509	2,569,385	rs587784256
c.288_289dup (p.Arg97fs)	R97fs	frame shift	VCV000211821	2,570,378—2,570,379	rs797045858
c.371 T > A (p.Val124Asp)	V124D	missense	VCV000159515	2,570,464	rs587784261
c.386A > T (p.Asp129Val)	D129V	missense	VCV000159517	2,570,479	rs587784263
c.399 + 1G > A		splice donor	VCV000159519	2,570,493	rs587784264
c.405G > A (p.Trp135Ter)	W135*	stop gain	VCV000159521	2,573,462	rs587784266
c.455_456del (p.Ser152fs)	S152fs	frame shift	VCV000159523	2,573,510—2,573,511	rs587784268
c.460C > T (p.Gln154Ter)	Q154*	stop gain	VCV000159524	2,573,517	rs587784269
c.484G > A (p.Gly162Ser)	G162S	missense	VCV000008079	2,573,541	rs121434487
c.524_528del (p.Lys175fs)	K175fs	frame shift	VCV000159526	2,573,579—2,573,583	rs587784270
c.537dup (p.Gln180fs)	Q180fs	frame shift	VCV000211826	2,573,590—2,573,591	rs587784271
c.537del (p.Gln180fs)	Q180fs	frame shift	VCV000159527	2,573,591	rs587784271
c.632C > G (p.Ser211Ter)	S211*	stop gain	VCV000159530	2,576,012	rs587784273
c.644_651del (p.Thr215fs)	T215fs	frame shift	VCV000159531	2,576,018—2,576,025	rs587784274
c.647_648del (p.Ile216fs)	I216fs	frame shift	VCV000159532	2,576,025—2,576,026	rs587784275
c.658del (p.Glu220fs)	E220fs	frame shift	VCV000159534	2,576,036	rs587784277
c.657G > A (p.Trp219Ter)	W219*	stop gain	VCV000159533	2,576,037	rs587784276
c.667dup (p.Thr223fs)	T223fs	frame shift	VCV000211828	2,576,044—2,576,045	rs797045864
c.664C > T (p.Gln222Ter)	Q222*	stop gain	VCV000159535	2,576,044	rs587784278
c.671G > A (p.Gly224Asp)	G224D	missense	VCV000159538	2,576,051	rs587784281
c.675C > G (p.Tyr225Ter)	Y225*	stop gain	VCV000159539	2,577,357	rs587784282
c.728_732dup (p.Asp245fs)	D245fs	frame shift	VCV000211831	2,577,406—2,577,407	rs797045867
c.730C > T (p.Gln244Ter)	Q244*	stop gain	VCV000159544	2,577,412	rs587784286
c.773_774del (p.Val258fs)	V258fs	frame shift	VCV000211833	2,577,453—2,577,454	rs797045869
c.829dup (p.His277fs)	H277fs	frame shift	VCV000211834	2,577,510—2,577,511	rs797045870
c.841 T > C (p.Cys281Arg)	C281R	missense	VCV000159546	2,577,523	rs587784288
c.851G > A (p.Trp284Ter)	W284*	stop gain	VCV000159548	2,577,533	rs587784289
c.900 + 1G > A		splice donor	VCV000159549	2,577,583	rs587784290
c.933dup (p.Leu312fs)	L312fs	frame shift	VCV000211836	2,579,830—2,579,831	rs797045872
c.1002 + 1G > A		splice donor	VCV000021175	2,579,901	rs113994203
c.1002 + 5G > A		non coding	VCV000159486	2,579,905	rs587784235
c.1003-30_1032del		splice acceptor	VCV000211817	2,583,426—2,583,485	rs1555527743
c.1009C > T (p.His337Tyr)	H337Y	missense	VCV000159489	2,583,464	rs587784236
c.1018dup (p.Trp340fs)	W340fs	frame shift	VCV000211818	2,583,472—2,583,473	rs797045855
c.1024_1031del (p.Arg342fs)	R342fs	frame shift	VCV000159490	2,583,479—2,583,486	rs587784237
c.1050dup (p.Lys351fs)	K351fs	frame shift	VCV000021177	2,583,499—2,583,500	rs113994200
c.1063del (p.Ser355fs)	S355fs	frame shift	VCV000159491	2,583,518	rs587784238
c.1064G > A (p.Ser355Asn)	S355N	missense	VCV000159492	2,583,519	rs587784239
c.1100del (p.Tyr367fs)	Y367fs	frame shift	VCV000159493	2,583,555	rs587784240
c.1135C > T (p.His379Tyr)	H379Y	missense	VCV000159495	2,583,590	rs587784242
c.1159G > T (p.Asp387Tyr)	D387Y	missense	VCV000159497	2,583,614	rs587784244
c.1165C > T (p.His389Tyr)	H389Y	missense	VCV000159498	2,585,028	rs587784245
c.1196G > C (p.Ser399Thr)	S399T	missense	VCV000159501	2,585,059	rs587784248
c.1201G > C (p.Asp401His)	D401H	missense	VCV000159502	2,585,064	rs587784249
c.1233A > C (p.Ter411Cys)		stop lost	VCV000159504	2,585,096	rs587784251
c.1111C > T (p.Arg371Ter)	R371*	stop gain	VCV000159494	2,583,566	rs587784241
c.568 + 1G > A		splice donor	VCV000436137	2,573,626	rs1555526733
c.162del (p.Lys54fs)	K54fs	frame shift	VCV000021180	2,569,347	rs113994198
c.265C > T (p.Arg89Ter)	R89*	stop gain	VCV000159512	2,570,358	rs587784258
c.817C > T (p.Arg273Ter)	R273*	stop gain	VCV000008074	2,577,499	rs121434483
c.305dup (p.Tyr102Ter)	Y102*	stop gain	VCV000159513	2,570,397—2,570,398	rs587784259
c.347dup (p.His117fs)	H117fs	frame shift	VCV000211823	2,570,436—2,570,437	rs797045859
c.368 T > A (p.Met123Lys)**	M123K	missense	VCV001077134	2,570,461	
c.523A > T (p.Lys175Ter)	K175*	stop gain	VCV000209180	2,573,580	rs797045061
c.910del (p.Ser304fs)	S304fs	frame shift	VCV000159551	2,579,802	rs587784292
c.911del (p.Ser304fs)	S304fs	frame shift	VCV000211835	2,579,809	rs797045871
c.852G > A (p.Trp284Ter)	W284*	stop gain	VCV000561072	2,577,534	rs1567559851
c.514dup (p.Met172fs)	M172fs	frame shift	VCV000436136	2,573,570—2,573,571	rs1555526718
c.430C > T (p.Arg144Ter)	R144*	stop gain	VCV000159522	2,573,487	rs587784267
c.1159 + 1G > A		splice donor	VCV000379162	2,583,615	rs1057520515
c.1159 + 2 T > A		splice donor	VCV000159496	2,583,616	rs587784243
c.569-10 T > C		non coding	VCV000021182	2,575,939	rs113994202
c.681dupG	L228Glufs	frame shift			this paper

Here, we comment on the case of a seven years old boy with an undiagnosed rare disease, with non-specific symptoms that could be compatible with LIS, but with an unclear presentation. Whole genome sequencing (WGS) of the patient was performed in the context of a genomics project (urugenomes.org) and sequence data was analyzed following a bioinformatics pipeline which concluded with a set of annotated and prioritized variants. A novel candidate frameshift variant was found that fits with the boy’s phenotype. To support the pathogenicity of the variant we used computational prediction tools and made segregation analysis with Sanger sequencing. To the best of our knowledge this is the first time this variant is reported [11] and it is the most likely cause of the patient’s disease.

## Case presentation

The index case is a seven years old boy with perinatal clinical records of poorly controlled pregnancy, homelessness and multiple drugs abuse through all gestation. Both parents are healthy and non-consanguineous. Early term delivery, low birthweight with microcephaly at birth with a head circumference of 32 cm (Z score -3).

The patient develops a spastic bilateral cerebral palsy, Gross Motor Function Classification System (GMFSC) IV, Bimanual fine motor function (BFMF) IVa, Communication Function Classification System (CFCS) IV. Associated with a profound intellectual disability, visual and auditory sensory deficit, pharmacoresistant epilepsy with generalized tonic clonic seizures, and congenital microcephaly with a head circumference growth always under -3 standard deviations. No dysmorphic signs were detected.

**Fig. 1:**
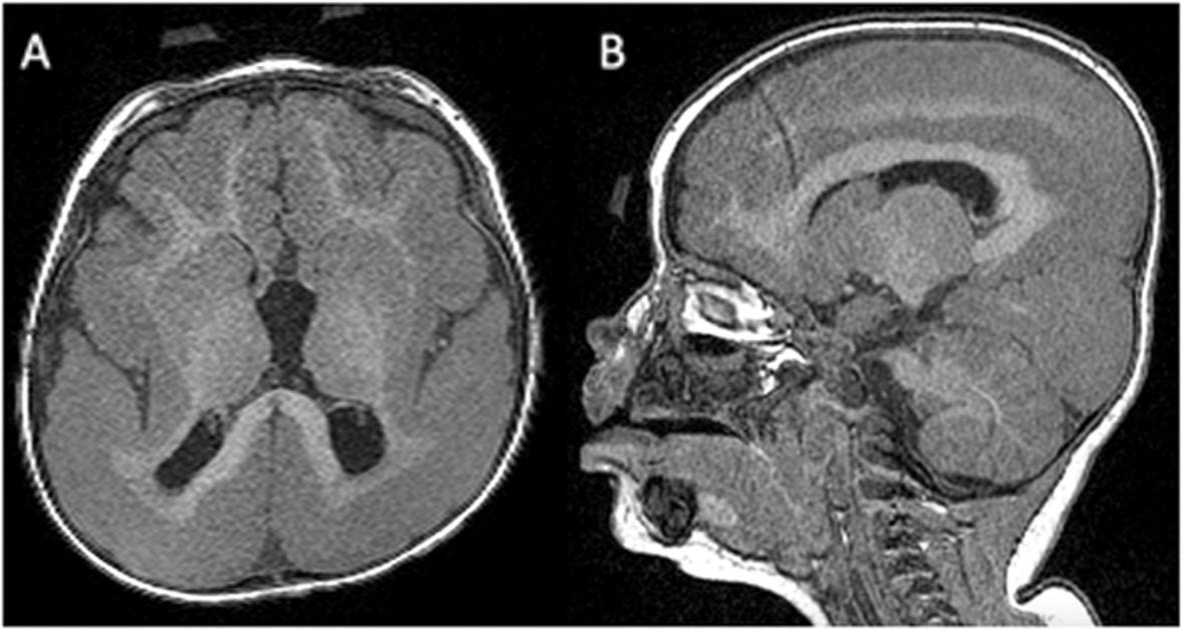
MRI results. **A** Volumetric T1 brain MRI axial and **B** Sagittal planes show a diffuse lissencephaly-pachygyria spectrum predominantly in the posterior areas methylation study for Angelman syndrome was normal and the sequencing of *ARX* and *MECP2* showed no pathogenic mutations

Cerebral magnetic resonance imaging (MRI) shows a diffuse lissencephaly-pachygyria spectrum with main affectation at posterior brain areas (Fig. 1).

## Genetic and evolutionary analysis

Whole genome sequencing (WGS) of the patient was performed and sequence data was analyzed following a bioinformatic pipeline which included analysis of the quality of reads [12], mapping onto human reference genome (hg19) [13], mark of duplicates, sorting and variant calling [14]. The variants obtained were annotated [15] and then subjected to different sets of filters to detect potentially causative mutations (see Supplementary Material). After these filters were applied, we obtained 40 homozygous or hemizygous variants with population frequency less than 1% located at splicing sites or coding regions, 458 heterozygous variants with population frequency less than 1% and 439 heterozygous variants with population frequency less than 0.5% and located at splicing sites or coding regions.

Among these prioritized variants we found a potential causative mutation in heterozygous state in the *LIS1/PAFAH1B1* gene. This gene was previously associated with the phenotype (LIS), especially with an autosomal dominant mechanism of inheritance. The potential causative mutation found is a frameshift insertion of a single nucleotide in exon 8 (PAFAH1B1:NM_000430:exon8:c.681dupG:p.(Lys228Glufs*28)), that lies between the first 23% to 55% of the protein depending on the transcript, according to the SIFT Indel tool [16]. The frameshift indel was reported as damaging with a confidence score of 0.858, and causing a nonsense mediated decay (NMD) response. It generates a premature stop codon 27 amino acids later, causing the loss of 156 amino acids.

If the mutated gene evaded NMD and led to a final product, this would be a 254 amino acids protein instead of the wild type 410 residues. A crystal structure has been described for LIS1 complexed to the brain cytosolic PAF-AH [17]. The complex shows that LIS1 folds into a beta propeller and interacts as a homodimer with a PAF-AH homodimer. From 14 reported surface interacting residues with PAF-AH, 8 are missing from our patient’s hypothetical protein. We predict that the mutated LIS1 could have self-aggregation tendencies, as the 27 new residues composing the shorter C-terminal region, not only would not allow the correct folding into a complete beta-propeller, but in addition would be highly disordered. As a qualitative indicator for this, the homology model in this C-terminal region has very low quality, in particular for the ‘HRTQRMGTY’ amino acid stretch, according to QMEANDiscO scoring function [18]. Even without aggregation and assuming the protein could fold into a ‘half propeller’, this protein would be unable to productively interact with PAF-AH as well as its additional molecular partners, notably dynein and a number of dynein-associated proteins. Indeed, LIS1 has been described as a molecular hub at a crossroad of several pathways, coupling PAF signaling to dynein regulation [17]. We expect all these functions to be hampered or inexistent in the protein product coded by this allelet.

The unaffected mother was sequenced at the proposed variant position and no mutation was detected. This is considered to support the hypothesis of a de novo mutation in the patient. Unfortunately the father of the patient (also unaffected) was not available for analysis.

**Fig. 2:**
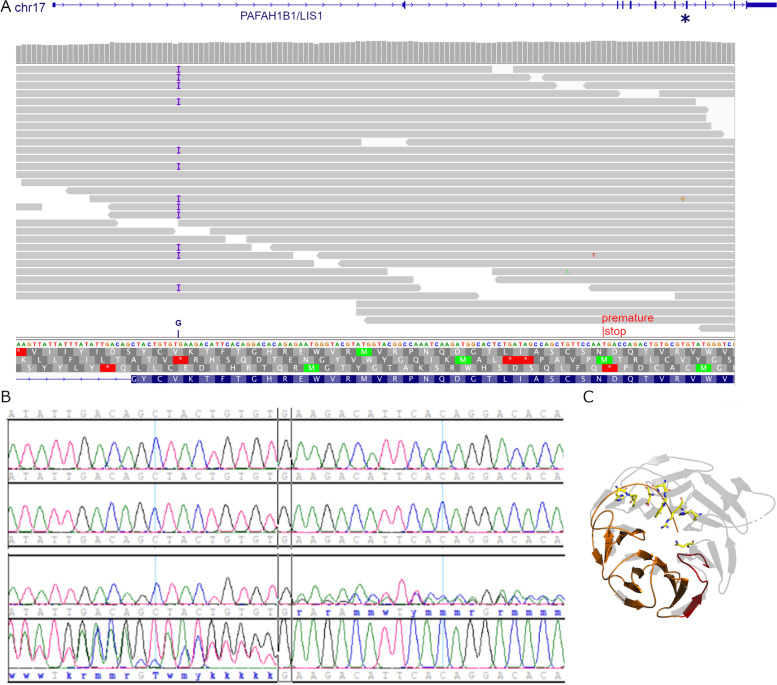
Variant impact at genome and protein level. **A** IGV view of the reads mapping onto the gene *PAFAH1B1*. The gene’s structure is shown on top and the exon, where the mutation is located is marked with an asterisk. The reads are shown in gray and the Insertion is marked in violet. The inserted G is shown on top of the reference genome. The originated premature stop codon is also marked. **B** Sanger sequencing of the variant in mother (top) and patient (bottom). Mother presents no variant, while patient variant was confirmed. **C** Top view of the truncated LIS1 beta propeller in orange. The C-terminal region of 27 residues introduced between the frameshift and the stop codon is painted in red. For reference, the wild-type crystal structure of LIS1 (PDB access code 1VYH) is shown superimposed as a transparent grey cartoon. Also, eight crucial interacting residues  are indicated in atom sticks, completely absent from the hypothetical truncated LIS1

Figure 2 A shows an IGV view of the candidate position. 37 reads are covering that location with a good quality. Additionally, the variant was confirmed with Sanger Sequencing in the patient (Fig. 2B, top) and was not seen in the mother (Fig. 2B, bottom)

## Discussion and conclusion

We found a novel probably causative frameshift variant in a patient with a previously undiagnosed rare disease using WGS. Previous genetic tests (sequencing of *MECP2* and *ARX* genes, and methylation analysis for Angelman syndrome) were performed with inconclusive results. This is expected since Lissencephaly and epileptic encephalopathy are highly heterogeneous genetic disorders in their etiology: ie. different genes are associated with several presentations of this pathology. For example, *RELN* gene is affected in the Norman-Roberts syndrome (LIS2) [19], heterozygous mutations in *TUBA1A* are responsible for the LIS3 syndrome [20], homozygous mutations in the *NDE1* gene are associated to LIS4 [21], among many others (*LAMB1* to LIS5 [22], *KATNB1* to LIS6 [23], *CDK5* to LIS7 [24], *TMTC* to LIS8 [25], *MACF1* LIS9 [26], *CEP85L* LIS10 [27]). Additionally X-linked forms of Lissencephaly are caused by *DCX* and *ARX* genes [28]. Hence, usually WGS or WES are accurate strategies for assessing patients with epileptic encephalopathy. However, in this case if we had had the MRI results (fairly consistent with LIS) before we had done the NGS sequencing, we might have end up doing a targeted sequencing approach, such as the *PAFAH1B1* gene or at least a subset of genes or WES, instead of doing the complete genome. This being a matter of costs and resources and not crucial for the patient’s diagnosis.

The variant we detected was an insertion of one nucleotide (G) in the coding sequence of *LIS1* gene, causing a change in the reading frame. The localization of the variant corresponds to the first 23% to 55% of the protein (depending on the transcript) and as a consequence a premature stop codon is produced causing the loss of the last 156 amino acids of the protein. Therefore, a severe affectation of the protein function is expected and probably a silencing of this allele either by encoding a truncated protein or by the mechanism of degradation of messenger RNA mediated by terminator mutations (NMD).

This variant has not been previously described and does not appear in the population frequency databases. It corresponds to the phenotype of the patient and the loss of function in the gene product is a mechanism already described in this condition (truncating mutations were described in other patients and being the gene involved in the microdeletion of Miller-Dieker lissencephaly syndrome). Since there are no other variants in the *LIS1/PAFAH1B1* gene with less than 1% population frequency and due to family history, we proposed a de novo mechanism for this case. This was (partially) confirmed by Sanger sequencing of the mother who doesn’t have the mutation. Father was unavailable for analysis, so this aspect remains unknown. Nevertheless, we consider that there is sufficient evidence that supports the pathogenic classification of the novel variant.

According to ACMG (American College of Medical Genetics and Genomics) variant interpretation guidelines [29] the frameshift variant found corresponds to the PVS1 (pathogenicity very strong) rule. It is a null variant (frameshift) in a gene where loss of function (LOF) is a known mechanism of disease (in ExAC database *PAFAH1B1* gene has a maximal probability of being LOF intolerant, pLI = 1 [30]). The frameshift mutation is also classified as a PM2 (moderate evidence of pathogenicity) since it was absent in population databases (1000 Genomes Project, GnomAD, etc.). We also consider applying rule PP4 (Patient’s phenotype is highly specific for a disease with a single genetic etiology), since the MRI findings are very specific for PAFAH1B1-related LIS. According to ACMG rules, the variant is classified as pathogenic, as it belongs to one very strong (PVS1), one moderate (PM2) and one supporting category (PP4).

We could also consider applying PP3 (supporting evidence of pathogenicity) since the pathogenic computational verdict is based on one pathogenic prediction from SIFT Indel Tool, one pathogenic prediction from the conservation score GERP [31] and no benign predictions. However, some studies avoid [32] applying PP3 in LoF variants when PVS1 is valid.

Furthermore there are other disruptive (frameshift or stop codon) variants in the same gene region reported as pathogenic [33], supporting the importance of the region for proper gene product function.

Through this work we were able to find a molecular diagnosis of a rare disease in a seven years old boy with severe and heterogeneous neurological symptoms. We found a *de novo* novel frameshift mutation in the *LIS1/PAFAH1B1* gene that most likely causes a silencing of one allele. This finding shows the benefit of the use of NGS as a diagnosis tool of rare diseases.

## Supplementary Information

**Additional file 1.**

## Data Availability

The variant found in this study is available in ClinVar, ID: SUB9799916 [11]. The bam files generated and analyzed during the current study are only available from the corresponding author upon reasonable request.

## Abbreviations

LIS: Lissencephaly
MCD: Malformations of cortical development
PAFAH1B1: Platelet-activating factor acetylhydrolase brain isoform Ib
PAF: Platelet activating factor
PAF-AH: Platelet-activating factor acetylhydrolase
WGS: Whole Genome Sequencing
GMFSC: Gross Motor Function Classification System
BFMF: Bimanual fine motor function
CFCS: Communication Function Classification System
MRI: Magnetic Resonance Imaging
ARX: Aristaless Related Homeobox
MECP2: Methyl-GpG binding protein 2
NGS: Next Generation Sequencing
NMD: Nonsense Mediated Decay
