# Supplementary material for: Novel frameshift mutation in LIS1 gene is a probable cause of lissencephaly: a case report
Source: BMC Pediatr. 2022 Sep 14;22:545. doi: 10.1186/s12887-022-03595-6 (PMC9472359; doi:10.1186/s12887-022-03595-6)
Supplement: Supplementary file 1 — Additional file 1. [file 12887_2022_3595_MOESM1_ESM.pdf]

Supplementary material

Methods

NGS sequencing and bioinformatics analysis

We carried out the WGS of the patient with paired end reads protocol on a Hiseq X ten Illumina sequencer (30x, 150PE), with an average depth of ~70x. 742.809.476 reads were obtained that passed the QC-controls and 711,616,662 reads (95.80%) were mapped onto the reference genome (GRCh37). Quality of reads was analyzed using FastQC [11], and they were mapped onto the human reference genome (GRCh37) using BWA [12]. Only unique reads mapping in proper pairs were further considered. Variant calling was performed using GATK (best practices) [13] and ANNOVAR [14] was used for the annotation process. 4.683.462 variants were detected in variant calling analysis, which were later annotated and prioritized. Different sets of filters were used in order to detect potentially causative mutations:

i. Homozygous mutations in coding/splicing regions with a population frequency lower than 1%;

ii. Heterozygous mutations in coding/splicing regions with at least two variants in the same gene and a population frequency lower than 1% (compound heterozygous);

iii. Heterozygous mutations in coding/splicing regions with a population frequency less than 0,5%;

iv. Mitochondrial variants: mutations with high heteroplasmy (>10%) and in coding regions or tRNA and rRNA genes (and not part of the definition of the haplogroup), not in D-Loop region.

Candidate frameshift mutation was further evaluated with the SIFT Indel tool [15], in order to estimate its pathogenicity effect.

Additionally, the mitochondrial genome was analyzed using MToolBox, for mapping, haplogroup prediction, variant calling and annotation, and heteroplasmy estimation [1].

Sanger sequencing was used to confirm the mutations in the index case and to analyse his mother.

Mitochondrial variants

Table 1: Variants prioritized in mitochondrial DNA. 37 variants prioritized in mitochondrial DNA analysis (of 139 variants detected) which had a significant heteroplasmy (greater than 10%), and from those only two were not a part of the definition of haplogroup. Those two had high levels of heteroplasmy but were synonymous mutations with no prediction of pathogenicity according to in silico scores (polyphen, SIFT) and lied in less conserved regions according to phyloP.

Variant Allele	Locus	Heteroplasmic Fraction	CI_lower.CI_upper	Haplogroup	Other Haplogroups	Aa.Change	PhyloP20Way
5156G	MT-ND2	1	0.998;1.0			syn	-0.653866
13713A	MT-ND5	0.999	0.997;1.0			syn	-11.805
4655A	MT-ND2	0.999	0.997;1.0		+	syn	-0.927669
6734A	MT-CO1	0.993	0.988;0.996		+	syn	-0.204937
15924G	MT-TT	1	0.998;1.0		+		-0.0232441
6674C	MT-CO1	0.995	0.991;0.997	U5b1i	+	syn	-4.8794
15777A	MT-CYB	1	0.998;1.0	U5b1i	+	S344N	-3.49534
3498T	MT-ND1	0.999	0.997;1.0	U5b1i		syn	-2.38007
7768G	MT-CO2	1	0.997;1.0	U5b1i	+	syn	-2.39326
14182C	MT-ND6	0.996	0.992;0.998	U5b1i	+	syn	-6.48914
13617C	MT-ND5	1	0.997;1.0	U5b1i	+	syn	-7.89333
9477A	MT-CO3	1	0.998;1.0	U5b1i	+	V91I	-1.25795
13276A	MT-ND5	1	0.998;1.0	U5b1i	+	V314M	-0.0522441
4312C	MT-TI	1	0.998;1.0	U5b1i	+		-0.467512
10664C	MT-ND4L	0.999	0.996;1.0	U5b1i	+	syn	-1.23228
10915T	MT-ND4	1	0.997;1.0	U5b1i	+	syn	-4.05698
7146A	MT-CO1	0.996	0.992;0.998	U5b1i	+	A415T	-2.98972
8468C	MT-ATP8	1	0.998;1.0	U5b1i	+	syn	-5.93247
13506C	MT-ND5	1	0.997;1.0	U5b1i	+	syn	-0.170291
8655C	MT-ATP6	1	0.998;1.0	U5b1i	+	syn	-1.98346
10688G	MT-ND4L	1	0.998;1.0	U5b1i	+	syn	-2.84639
10810T	MT-ND4	1	0.998;1.0	U5b1i	+	syn	-7.78961
4104A	MT-ND1	1	0.998;1.0	U5b1i	+	syn	-0.352472
7256C	MT-CO1	1	0.998;1.0	U5b1i	+	syn	-2.39326
3594C	MT-ND1	1	0.998;1.0	U5b1i	+	syn	-3.58527
13650C	MT-ND5	1	0.998;1.0	U5b1i	+	syn	-0.37089
7521G	MT-TD	0.997	0.993;0.999	U5b1i	+		-2.13217
13105A	MT-ND5	0.995	0.991;0.997	U5b1i	+	V257I	-0.634669
11914G	MT-ND4	1	0.997;1.0	U5b1i	+	syn	-0.379976
12308G	MT-TL2	0.999	0.996;1.0	U5b1i	+		http://mamit-trna.u-strasbg.fr/mutations.asp?idAA=17
11467G	MT-ND4	1	0.997;1.0	U5b1i	+	syn	-1.10246
12372A	MT-ND5	0.999	0.996;1.0	U5b1i	+	syn	-7.98779
9540T	MT-CO3	1	0.997;1.0	U5b1i	+	syn	-4.15935
10873T	MT-ND4	1	0.997;1.0	U5b1i	+	syn	-1.43405
8701A	MT-ATP6	1	0.998;1.0	U5b1i	+	A59T	-2.15897
12705C	MT-ND5	0.996	0.992;0.998	U5b1i	+	syn	-3.91081
10398A	MT-ND3	1	0.998;1.0	U5b1i	+	A114T	-4.66227

Non-coding variants

Additionally we detected 178067 non-coding variants. Among the non-coding variants we prioritized those previously submitted to ClinVar as pathogenic (including the labels “Pathogenic”, “Likely pathogenic”, “Pathogenic, protective” and “Pathogenic, risk factor” or

“conflicting interpretations of pathogenicity”) or as uncertain significance. Using these last filters it was obtained 57 variants reported with uncertain significance, 4 variants with conflicting interpretations of pathogenicity and no pathogenic reported variants.

PAFAH1B1 mutations reported in ClinVar associated to cortical brain malformation in children

To gather information regarding mutations associated with cortical brain malformations in children (including lissencephaly) we analyzed the ClinVar database [2] accessed on the 18th April 2022. We downloaded all 336 mutations in the PAFAH1B1 gene with at least one star in the database (variants with a minimum amount of evidence). We only report the ones with labels Likely Pathogenic or Pathogenic (148). Of those, we report the 101 mutations (Table 1) associated with the specific phenotype (mutations with not provided phenotype were filtered out). Of the 101 mutations, 31 were missense, 32 frame shift, 6 non-coding, 19 stop gain, 11 splicing (9 donor, 2 acceptor site), 2 large deletions.

References supplementary material

[1] Calabrese C, Simone D, Diroma MA, Santorsola M, Gutta C, Gasparre G, et al. MToolBox: A highly automated pipeline for heteroplasmy annotation and prioritization analysis of human mitochondrial variants in high-throughput sequencing. Bioinformatics. 2014;30(21):3115–7. https://doi.org/10.1093/ bioinformatics/btu483.

[2] Landrum, M. J., Lee, J. M., Benson, M., Brown, G., Chao, C., Chitipiralla, S., ... & Maglott, D. R. (2016). ClinVar: public archive of interpretations of clinically relevant variants. *Nucleic acids research*, *44*(D1), D862-D868.
